# DNA Barcode Reveals the Bycatch of Endangered Batoids Species in the Southwest Atlantic: Implications for Sustainable Fisheries Management and Conservation Efforts

**DOI:** 10.3390/genes10040304

**Published:** 2019-04-18

**Authors:** Bruno Lopes da Silva Ferrette, Rodrigo Rodrigues Domingues, Matheus Marcos Rotundo, Marina Provetti Miranda, Ingrid Vasconcellos Bunholi, Juliana Beltramin De Biasi, Claudio Oliveira, Fausto Foresti, Fernando Fernandes Mendonça

**Affiliations:** 1 Laboratório de Genética e Conservação, Universidade Santa Cecília (UNISANTA), Santos 11045-907, Brazil; 2 Laboratório de Genética Pesqueira e Conservação (GenPesC), Instituto do Mar, Universidade Federal de São Paulo (UNIFESP), Santos 11070-102, Brazil; domingues.pesca@gmail.com (R.R.D.); marinaprovetti@gmail.com (M.P.M.); inngridbunholi@gmail.com (I.V.B.); jubbiasi@gmail.com (J.B.D.B.); fernando.mendonca@unifesp.br (F.F.M.); 3 Acervo Zoológico, Universidade Santa Cecília (UNISANTA), Oswaldo Cruz St. 266, Santos 11045-907, Brazil; mmrotundo@unisanta.br; 4 Laboratório de Biologia e Genética de Peixes (LBGP), Instituto de Biociências de Botucatu (IBB), Universidade Estadual Paulista “Júlio de Mesquita Filho” (UNESP), Botucatu 18618-689, Brazil; claudio@ibb.unesp.br (C.O.); f.foresti@unesp.br (F.F.)

**Keywords:** genetic identification, bycatch composition, threatened species, molecular marker, conservation

## Abstract

Today, elasmobranchs are one the most threatened vertebrate groups worldwide. In fact, at least 90% of elasmobranch species are listed in the International Union for Conservation of Nature (IUCN) Red List, while more than 40% are data-deficient. Although these vertebrates are mainly affected by unsustainable fishery activities, bycatch is also one of the major threats to sharks and batoids worldwide, and represents a challenge for both sustainable fishery management and for biodiversity and conservational efforts. Thus, in this study, DNA barcode methodology was used to identify the bycatch composition of batoid species from small-scale industrial fisheries in the southwest Atlantic and artisanal fisheries from southeast Brazil. A total of 228 individuals belonging to four Chondrichthyes orders, seven families, and at least 17 distinct batoid species were sequenced; among these individuals, 131 belonged to species protected in Brazil, 101 to globally threatened species, and some to species with trade restrictions provided by Appendix II of the Convention on International Trade in Endangered Species (CITES). These results highlight the impacts on marine biodiversity of bycatch by small-scale industrial and unmanaged artisanal fisheries from the southwest Atlantic, and support the implementation of DNA-based methodologies for species-specific identification in data-poor fisheries as a powerful tool for improving the quality of fisheries’ catch statistics and for keeping precise bycatch records.

## 1. Introduction

Batoidea is a superorder of cartilaginous fish commonly known as rays, stingrays, or skates (hereafter “batoids”). They are currently the largest subgroup of the Chondrichthyes class and include 26 families with 633 valid species, and at least 50 unrecognized or undescribed species [1,2]. Batoids are caught as bycatch in different fishing equipment worldwide [3]. As a consequence of the high exposure to fishing activity on the continental shelf, some batoid species are on the brink of extinction. In fact, five of the seven most endangered Chondrichthyes families worldwide are from the Batoidea superorder [4]. In 2018, the International Union for Conservation of Nature (IUCN) assessed a total of 573 batoids, of which ~21% are in the threatened categories, and 41.7% are data deficient [5].

According to the Food and Agriculture Organization (FAO) of the United Nations (UN), the reported global production of sharks and batoids in 2016 was more than 767,000 tons, and batoids accounted for ~36% of catches [6]. However, this does not reflect real catches because these reports neither include discard numbers nor illegal, unreported, and unregulated (IUU) fisheries, which has led to underestimation of the actual magnitude of the reported catches [7]. Most batoid captures consist of unreported bycatch from trawlers and gillnet fisheries [4]. According to the most popular definition, bycatch is the non-target marine animals caught unintentionally during fishing activities [8]. Bycatch is commonly consists of (a) non-target species that are kept and eaten/sold, and (b) discards, which are those organisms that are thrown back (alive or dead) into the sea. The discards are the common focus of studies on bycatch, as they are a subset that represents a waste of fishery resources and thus attracts significant public awareness, and usually includes endangered, threatened, or protected species [9].

Discards consist of those specimens that lack market demand, are physically damaged, have low trade value, exceed allowed quotas, or represent illegal retention onboard [10,11]. However, discards that have commercial value are usually unmanaged, because fisheries management focuses mostly on target species [12]. The poor management of non-target catches compromises sustainability efforts and can result in substantial undocumented biomass removal [13]. Trawlers and gillnets use non-selective gear and catch many non-target species. Indeed, these fishing techniques present high discard rates and are considered to be the most wasteful form of fishing, which affects elasmobranch as well [14,15]. The lack of available information on coastal trawling fisheries is of great concern given the number of elasmobranch bycatch [16], and thus the impact of fisheries emphasizes the need to assess not only the conservation status of target species, but also that of bycatch and discarded species. Thus, the collection of species-specific data on these fishing methods is of the utmost importance and should therefore be implemented [3].

Only a small percentage of batoids are properly reported at the lowest taxonomic level [12]. The lack of species-specific identification is a severe problem for fishing activity, hindering the efforts of fisheries management, as well as making it extremely hard to implement the supervision of protected and endangered species [17]. The inadequate identification of elasmobranch species, as well as their poor commercial labeling, are major concerns worldwide, as they undermine biodiversity conservation objectives designed to promote sustainability [18]. Nowadays, traditional morphological methods for species identification are coupled with DNA-based approaches (e.g., DNA barcoding) [19]. These techniques have undergone rapid progress, becoming widely applied mainly due to cost reductions combined with the need to address critical conservation issues and fisheries management problems [20]. Solutions based on scientific data have made molecular techniques applicable to several current biological issues, such as species identification, population structure, and the evaluation of fishery genetic stocks, as well as the evolutionary relationships among species [21].

Genetic approaches have a long and successful history when it comes to shark identification, as documented in many studies conducted worldwide for trade surveillance and biodiversity conservation purposes [22,23,24,25,26,27]. However, overall, batoids received less attention in research studies compared to sharks [28,29,30]. Within this context, our main objective was to identify bycatch batoid specimens caught by trawlers and gillnets from small-scale industrial and artisanal fisheries by applying DNA barcoding, in order to improve bycatch species-specific statistics and gather more biodiversity information on bycatch fauna from south–southeast of Brazil in the southwest Atlantic.

## 2. Materials and Methods

### 2.1. Sampling

**Figure 1:**
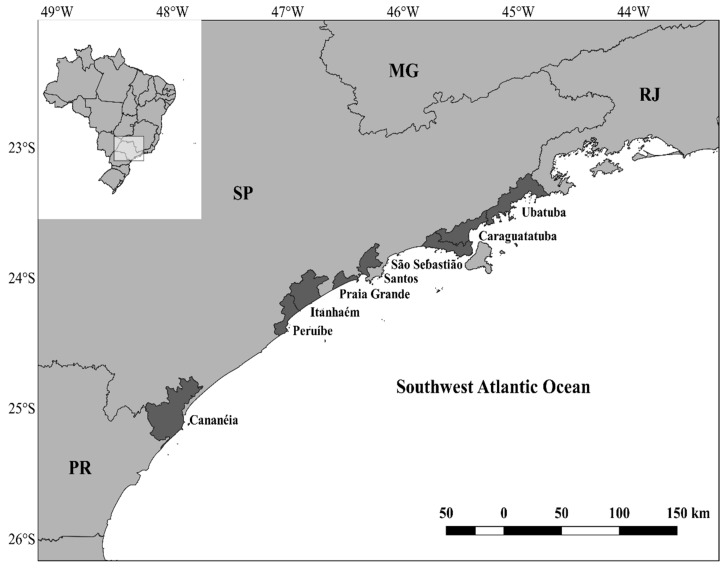
Batoid sampling locations scattered along the coast of the states of São Paulo (SP), Minas Gerais (MG), Rio de Janeiro (RJ), and Paraná (PR).

Batoid muscle tissue samples were obtained from bycatch specimens from artisanal fisheries, such as gillnets and fish-traps, and also small-scale trawler fisheries, such as otter-trawls and beam-trawls, operating along the Brazilian south–southeast coast between 2012 and 2018 (Figure 1). All tissues samples were stored in 95% ethyl alcohol at −20 °C and deposited at the collection of the Genetics Fisheries and Conservation Laboratory (GenPesC) at the Marine Institute (IMar) of the Federal University of São Paulo (UNIFESP), campus Baixada Santista, under a permanent license issued by the Brazilian Ministry of the Environment (No. 50463-1).

### 2.2. DNA Extraction, Amplification, and Sequencing

Genomic DNA was extracted using the NucleoSpin^®^ Tissue kit (MACHEREY-NAGEL GmbH & Co.). The mitochondrial cytochrome *c* oxidase subunit I gene (COI) was amplified by polymerase chain reaction (PCR) using the Platinum^®^ Taq DNA Polymerase kit (Invitrogen^™^) with the following primers: forward: Fish-F1: 5′-TCA ACC AAC CAC AAA GAC ATT GGC AC-3′, and reverse: Fish-R1: 5′-TAG ACT TCT GGG TGG CCA AAG AAT CA-3′ [31]. PCR products were purified with ExoSAP-IT^™^ PCR Product Cleanup Reagent (Applied Biosystems^™^). Sequencing reactions were carried out using the BigDye^™^ Terminator v3.1 Cycle Sequencing Kit (Applied Biosystems^™^), according to the manufacturer’s instructions, on a 3130xl Genetic Analyzer (Applied Biosystems^™^).

### 2.3. DNA-Based Species Identification

For taxonomic identification, no traditional morphological method was used due to the fact that the vast majority of individuals were not intact, which would compromise the identification itself. The species common names used by fishermen were not considered either, as they could bias identification; only DNA-based species identification was employed. Two methodological approaches were applied for species assignment: sequence similarity-based identification and phylogenetic tree-based identification [32]. First, a similarity test was performed between our sequences and the reference libraries in the genetic databases BOLD (the Barcode of Life Data system) [33] and GenBank^®^ [34] using similarity scores (e.g., BLAST) [35], assigning the species name to the sequence with the highest similarity. Second, a phylogenetic tree-based method [36] was executed to assign unidentified barcodes to species based on tree clusters to estimate the phylogenic relationship between the reference barcodes and the query sequence. The query was assigned to the species it clustered within.

The second approach involved the construction of a phylogenetic tree based on the maximum-likelihood (ML) method, which was built using PhyML 3.2.2 [37] and implemented on Geneious R11.1.5 [38]. The PhyML uses a BioNJ tree as a starting tree, and tree topology search was set as the best from the nearest neighbor interchange (NNI) and subtree pruning and regrafting (SPR) methods. Robustness of reconstructed trees was estimated with 1000 non-parametric bootstrap replicates [39]. All COI sequences were aligned using the Multiple Sequence Comparison by Log-Expectation (MUSCLE) algorithm [40] in Geneious R11.1.5. The COI matrix was tested for nucleotide substitution saturation [41] with DAMBE6 [42] and showed no significant saturation. The best-fit nucleotide substitution model, the Generalized Time-Reversible Model (GTR), with invariable sites and Gamma shape parameter alpha (GTR + I + G) (*p*-inv = 0.57, α = 1.528), was evaluated using the Bayesian Information Criterion (BIC) and the Decision Theory (DT) with jModelTest2 on XSEDE [43,44] through the CIPRES Science Gateway v3.3 [45]. The PhyML tree was set as the initial tree of the Bayesian inference performed in BEAST v1.10.2 on XSEDE [46], adopting an unregulated relaxed lognormal clock [47] and the Yule speciation process [48,49] as priors. The posterior probability of parameters was estimated using Markov chain Monte Carlo (MCMC) with 300 million generations and 10% of burn-in. The model convergence, effective sample size (ESS), and 95% credible HPD (Highest Posterior Density) intervals were all calculated using TRACER, v1.7.1 [50]. The software TreeAnnotator v1.10.2 summarized the information of a sample of trees produced by BEAST onto a single Highest Log Clade Credibility Tree. For the phylogenetic tree construction, the generated sequences were coupled with the highest similarity score sequences downloaded from the Barcode Index Numbers (BINs) [51] (Appendix A), except for the species *Rhinoptera bonasus*, for which the sequences were obtained only from GenBank^®^ because of the lack of a BIN. The American elephant fish, *Callorhinchus callorynchus* (FARG380-08) was defined as an outgroup.

## 3. Results

**Figure 2:**
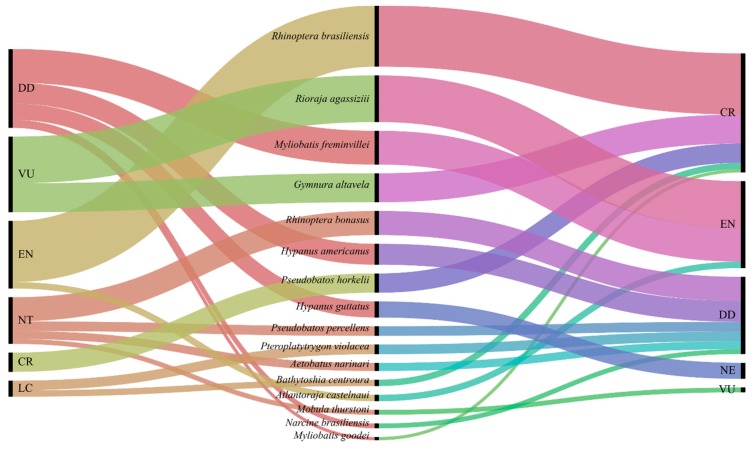
Alluvial diagrams allow to represent the correlation between the identified batoid species and their conservation status on the International Union for Conservation of Nature (IUCN) Red List and the Brazilian Red List. The left side of the chart shows the correlations between the identified batoid species and the IUCN Red List status; the right side shows the correlation between the identified batoids species and the Brazilian Red List. The chart was built using the online tool RAWGraphs (https://rawgraphs.io/).

A total of 652 base pairs (bp) of COI barcode from 228 individuals were successfully sequenced and identified to the species level, and the results showed a high similarity (98.0–100.0%) to the COI sequences from the genetic databases, allowing the identification of four orders (Myliobatiformes, Rajiformes, Rhinopristiformes, Torpediniformes), seven families (Dasyatidae, Myliobatidae, Rhinopteridae, Gymnuridae, Arhynchobatidae, Rhinobatidae, Narcinidae), and at least 17 distinct batoid species (Table 1). The species *Rhinoptera brasiliensis*, *Rioraja agassizii*, *Myliobatis freminvillei*, and *Gymnura altavela* were the most represented, comprising 46.49% of the total samples. Among the identified samples, 44.3% belonged to species listed on the threat categories of the IUCN Red List, while 57.47% were protected in Brazil under Ordinance No. 445, dated 17 December 2014, issued by the Department of the Environment (MMA). In addition to the high proportion of threatened species, a total of 21.5% “data-deficient” (DD) batoid species were, for which the lack of biological information makes it impossible to properly evaluate their extinction risks (Table 1, Figure 2). *Mobula thurstoni* (3 specimens found) is listed in Appendix II of Convention on International Trade in Endangered Species (CITES) owning trade regulations besides being protected in Brazil. All the generated sequences were submitted to the GenBank^®^ database under the accession numbers MK085522 to MK085749.

**Figure 3:**
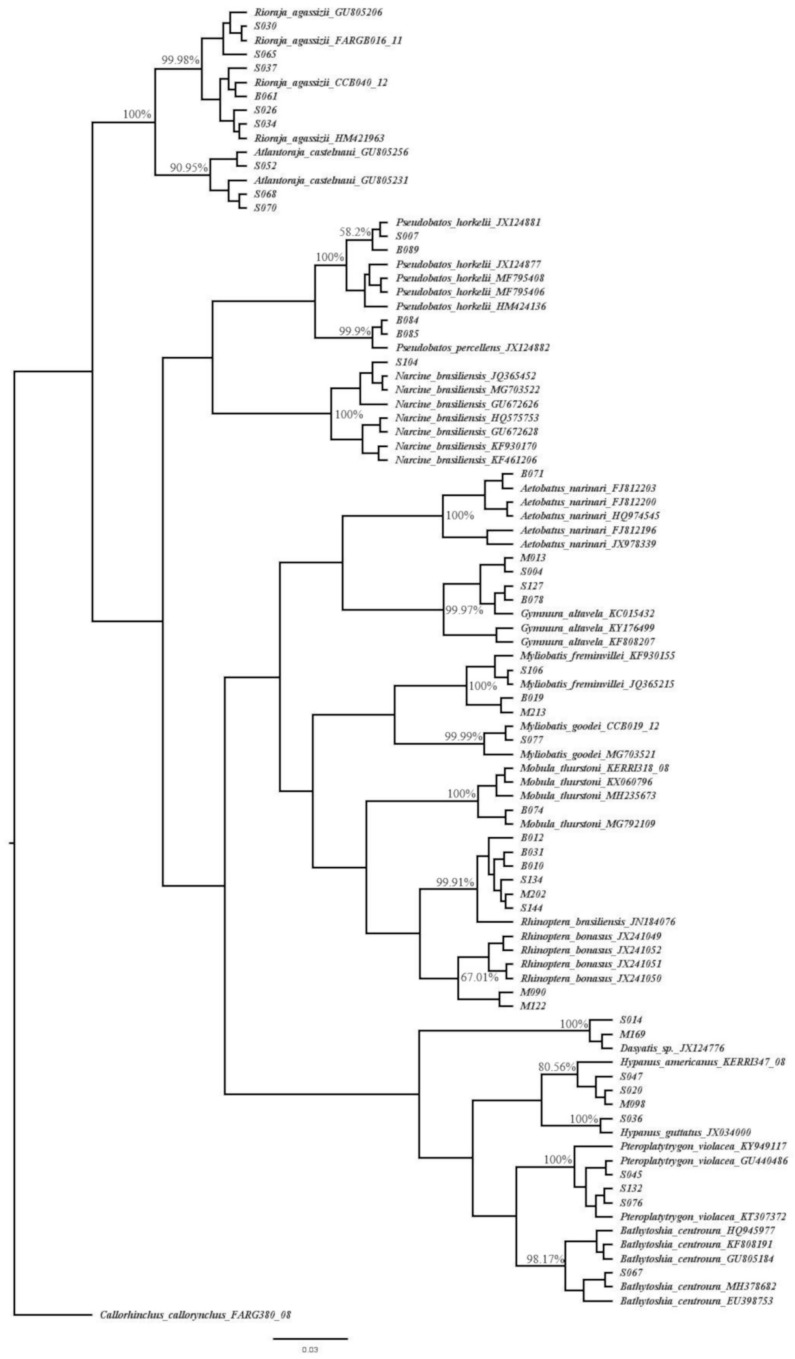
Bayesian phylogenetic inference tree of cytochrome *c* oxidase subunit I (COI) using 94 haplotypes with 652 base pairs of sequences from BOLD, GenBank^®^, and also from this study. Node values indicate the percentage of posterior credibility values of trees. The plausible phylogenetic tree for the given data was produced using the Yule speciation model based on prior probabilities. Sxxx, Bxxx and Mxxx codes comprise the sequenced batoids individuals from this study.

The Bayesian phylogenetic tree (Figure 3) was effective to separate all the identified sequence clusters from different species, since the topology of the phylogenetic tree, supported by high posterior credibility values, strengthened the sequence similarity-based identification, even for those that did not match at the lowest taxonomic level, such as *Dasyatis* sp. sequences, which accounted for 16.5% of the identified specimens (Table 1).

## 4. Discussion

The results demonstrate that the bycatch composition of batoid species in the central–southwest Atlantic consists of several protected species in Brazil, and also globally threatened species. According to the IUCN Red List, most of the identified species are classified as “data deficient” (DD), followed by “vulnerable” (VU) and “endangered” (EN) species (Figure 2). In addition, according to the Brazilian Red List, the majority of the identified species are classified as “critically endangered” CR and EN (Figure 2), and there is even a species, *Mobula thurstoni*, listed in Appendix II of CITES, which includes species that are not necessarily threatened with extinction, but whose trade must be regulated in order to avoid unsustainable exploration. Besides CITES regulations, mobulids have been reported worldwide as bycatch in several small- and large-scale fisheries [52], and have been previously reported as bycatch in the southwestern Atlantic Ocean [53]. In addition, artisanal fishing can also have impacts on the population levels. Reference [54] attributed an 88% decline in the Mozambique population of *M. alfredi* between 2003 and 2011 mostly to artisanal fishing.

In general, high-seas fisheries present more detailed and reliable elasmobranch bycatch data than coastal fisheries, probably because coastal fisheries are more diverse and complex [55].

According to the monitoring program for fishing activity of the state of São Paulo, during the study period, elasmobranch catches totalized 1,952,625.39 tons and showed an evident decrease in productivity over the years. If we consider only the sampled fisheries, which accounted for 69.59% of elasmobranch captures in the whole state, they consist of gillnets (42.82%), followed by otter-trawl (36.34%), beam-trawl (20.01%), and fish-traps (0.83%), (Appendix A). The development of a precise catch-data information system for fisheries that recognizes the peculiarities of this sector takes a huge effort. One of the main reasons for the lack of reliable and standardized fisheries data is related to the large variety of fishing gear, target species, and landing sites, which end up biasing catch-data sampling methods [56]. In addition, fishery catch trends often lack species-specific data, and in most cases, discards are neither recorded nor reported [57]. Such a situation has led to local extinctions of skates due to trawling, as occurred, for example, in the Irish Sea [58] and northwest Atlantic [59]. The impact of fisheries on biodiversity highlights the need to assess not only the conservation status of target species, but also of bycatch species, in particular for batoids discarded in coastal trawl fisheries. A high proportion of batoid bycatch, which fluctuated seasonally between 44.5% and 67.5% of the total trawlers’ capture biomass, was documented in Argentina [60], which is also located in the southwest Atlantic.

Bycatch research has traditionally evaluated large-scale fisheries, neglecting the potentially harmful effects of small-scale and artisanal fisheries on threatened species [61]. Nevertheless, it is of the utmost importance to encourage effective bycatch mitigation strategies for artisanal and small-scale fisheries, which account for the vast majority of the fishing workforce worldwide and mainly operate in coastal and continental shelf regions, which feature high productivity and aggregate high levels of marine biodiversity [61,62,63]. Some of the identified batoid species are widely distributed in the Atlantic, although most of them are endemic to the south–southwest Atlantic. According to Reference [64], restricted-range endemic species are particularly vulnerable, and, for this reason, conservation efforts must prioritize the identification of biodiversity hotspots based on species richness and endemism degree [65,66]. The southwest Atlantic is one of the most important and recognized global hotspots of elasmobranch species richness, functional diversity, and endemicity [4,67] and, as such, researchers, authorities, policy makers, and fisheries managers must prepare conservational plans focusing efforts and resources to preserve its biodiversity and mitigate the harmful effects of unsustainable fishing activity, such as bycatch. For instance, *Pseudobatos horkelli*, an endemic Brazilian guitarfish, is currently classified as CR by the IUCN Red List, and suffered a severe population decline of more than 80% due to intense anthropogenic pressures [68]. Another endemic skate, *Atlantoraja castenauai*, also suffered a massive population decline of about 75% [69]. On top of that, another endemic ray, the Brazilian cownose ray, *Rhinoptera brasiliensis*, was the most commonly identified species, accounting for 17.43% of total sampling. In fact, this species forms large groups and is susceptible to trawlers and gillnets that capture a large number of individuals, and, due to its life history, this ray is vulnerable to recruitment overfishing [70]. The same threats have already been observed in two endemic coastal sharks, the daggernose shark, *Isogomphodon oxyrhynchus*, from northern Brazil, and the spiny angel shark, *Squatina guggenheim*, from southern Brazil. Both species showed severe population declines, up to at least 90% and 85%, respectively, due to anthropogenic and fisheries threats similar to those described above [71,72,73]. This troubling scenario demonstrates the urgent need for improvements in Brazilian fisheries management in relation to Chondrichthyes, as observed by Reference [74].

This unsustainable fisheries situation is likely to stay unchanged or even escalate as unrestricted fishing continues without proper regulation, increasing the imminent risk of extinction for these species [69,70,72]. Although Brazilian law prohibits the capture, commercialization, and trade of many elasmobranch species, they are still illegally captured, landed, and traded [26,28].

Currently, 41.74% of the IUCN-listed elasmobranch species are classified as DD [5]. The lack of biological data is higher in chondrichthyans than in other evaluated vertebrate groups due to taxonomic uncertainties, incomplete range distributions, and biased population assessments. Our results showed that almost 22.47% of identified batoid species were classified as DD. These uncertainties can considerably influence the understanding of threats to these species and their risk patterns [75]. As a consequence, species potentially threatened could be currently neglected by conservation programs. Therefore, knowing the species’ actual conservation status is crucial for the implementation of proper biodiversity conservation efforts [76].

According to our results, 16.5% of samples did not match at the lowest taxonomic level, but only at the genus level, *Dasyatis*. This may happen if a species has not been properly deposited in genetic databases, or in the case of a non-formally described species or even an unknown species. However, the topology of the Bayesian phylogenetic tree highlights the clustering of the sequences identified as *Dasyatis* sp. on a separate clade, showing that individuals may belong to a different species but not to the other closely related identified species. This example has shown the importance of coupling the two methodological approaches used for species assignment in our study, sequence-similarity-based-identification and phylogenetic-tree-based-identification, since the Bayesian inference strengthened and conferred robustness upon the species identification, even in those cases where species-specific identification could not match with the lowest taxonomic level. Nevertheless, several batoid groups require taxonomic and nomenclature revisions in BOLD and GenBank^®^ databases. This is a critical problem, since the names assigned to the sequences need to be continuously updated, and there are still sequences identified only at the genus or family level, making them non-informative for molecular identification purposes [77]. The level of the batoids’ taxonomic uncertainties was highlighted by Reference [78], and recently, the taxonomy of the Dasyatidae family was reviewed by Reference [79] through morphological and molecular approaches.

The impossibility of properly identifying different captured species results in incomplete, unreliable, and underestimated fisheries catch statistics, which makes it difficult to manage fisheries’ activity in a sustainable way [80]. Neglecting small-scale fisheries worldwide results in misleading statistics reported annually by FAO members, which omit and/or underestimate statistical fisheries catch-data, such as discards and IUU fisheries [81]. Reference [7] concluded that global catches reported to FAO between 1950 and 2010 were actually 50% higher than reported, and catches also declined more intensively. Data on the identification and quantification of each of the captured species are the most basic information for effective fisheries management. Nevertheless, this information is unavailable in many regions worldwide, which makes the situation even worse because the absence of adequate identification can generate non-effective management measures, as observed in Australia [82], where it was demonstrated that “at-vessel mortality” of sharks and rays in the same type of fishing gear can largely vary among species. In many countries of the southwest Atlantic, such as Brazil, the collection system of statistical fishery data has fallen apart, meaning that currently there is no national standardized data system; in fact, the reconstructed Brazilian catches were 1.8 times larger than previously reported landings [83]. Minimizing fisheries bycatch is a global environmental challenge [84,85]. Although fisheries scientists, managers, and industry have made substantial progress in improving fisheries management in data-poor fisheries [86], and also mitigating interactions and/or reducing bycatch with particular focus on endangered, threatened, and protected species, they continually aim to improve the performance of bycatch mitigation strategies while maintaining sustainable commercial harvest levels [84,87,88].

In the last few years, several studies in Brazil have developed and implemented molecular markers to identified elasmobranchs [25,26,89,90]. However, overall, a few studies have been focused on batoids [28,77,91]. Such findings highlight the necessity to implement DNA-based approaches to improve fisheries species-specific catch-data, and also for trading surveillance purposes. Thus, DNA barcoding has emerged as a widely accepted tool for species identification, due to its improved focus on data standardization and validation [92]. To conclude, our results show the bycatch batoid species composition in the southwest Atlantic, which consist of some species under protection by national and international laws. In order to attenuate fishery impacts over biodiversity, managers should monitor and report catches at the species level, prohibit trade and landings of threatened and protected species, and manage bycatch to minimize mortality of non-target species with the adoption of new technologies aiming to mitigate bycatch [93,94,95]. Crucial to this process is the implementation of an accurate and standardized DNA-based species identification method, such as DNA barcoding, to improve and promote the sustainability of biodiversity, as well as to support the productivity of the fishery activity.

## Figures and Tables

**Table 1:** Identified batoid species; n, number of identified individuals; %, percentage of total sampling; IUCN, International Union for Conservation of Nature Red List species status; ICMBio, Brazilian Red List species status. CR, Critically Endangered; EN, Endangered; VU, Vulnerable; NT, Near Threatened; LC, Least Concern; DD, Data Deficient.

Order	Family	Common Name	Species	n	%	IUCN	ICMBio
Myliobatiformes	Dasyatidae	Roughtail stingray	*Bathytoshia centroura*	4	1.75%	LC	CR
		Southern stingray	*Hypanus americanus*	13	5.70%	DD	DD
		Longnose stingray	*Hypanus guttatus*	10	4.39%	DD	NE
		Blue pelagic stingray	*Pteroplatytrygon violacea*	6	2.63%	LC	DD
			*Dasyatis* sp.	39	17.11%	-	-
	Myliobatidae	Spotted eagle ray	*Aetobatus narinari*	5	2.19%	NT	DD
		Smoothtail mobula	*Mobula thurstoni*	3	1.32%	NT	VU
		Bullnose ray	*Myliobatis freminvillei*	21	9.21%	DD	EN
		Southern eagle ray	*Myliobatis goodei*	2	0.88%	DD	CR
	Rhinopteridae	Cownose ray	*Rhinoptera bonasus*	15	6.58%	NT	DD
		Brazilian cownose ray	*Rhinoptera brasiliensis*	38	16.67%	EN	CR
	Gymnuridae	Spiny butterfly ray	*Gymnura altavela*	18	7.89%	VU	CR
Rajiformes	Arhynchobatidae	Spotback skate	*Atlantoraja castelnaui*	4	1.75%	EN	EN
		Rio skate	*Rioraja agassizii*	29	12.72%	VU	EN
Rhinopristiformes	Rhinobatidae	Brazilian guitarfish	*Pseudobatos horkelii*	12	5.26%	CR	CR
		Southern guitarfish	*Pseudobatos percellens*	6	2.63%	NT	DD
Torpediniformes	Narcinidae	Brazilian electric ray	*Narcine brasiliensis*	3	1.32%	DD	DD
				228	100.00%		

## Appendix A

**Table A1:** GenBank sequences utilized for the construction of the phylogenetic tree with barcode index numbers (BINs) of the identified species.

Species	GenBank Access Number
*Atlantoraja castelnaui*	GU805239
*Atlantoraja castelnaui*	GU805231
*Atlantoraja castelnaui*	JX124737
*Atlantoraja castelnaui*	JQ365232
*Hypanus americanus*	KJ719465
*Hypanus americanus*	KT075327
*Hypanus americanus*	KF461168
*Bathytoshia centroura*	KT075311
*Bathytoshia centroura*	KJ719464
*Bathytoshia centroura*	KF808191
*Hypanus guttatus*	JX034000
*Hypanus guttatus*	KJ719467
*Dasyatis* sp.	JX124776
*Gymnura altavela*	KC015432
*Gymnura altavela*	BIM344-13
*Gymnura altavela*	BIM365-13
*Gymnura altavela*	KF808207
*Myliobatis freminvillei*	KT075326
*Myliobatis freminvillei*	KF930155
*Myliobatis goodei*	JQ305804
*Myliobatis goodei*	JQ305809
*Myliobatis goodei*	JQ305807
*Myliobatis goodei*	JQ305806
*Narcine brasiliensis*	JQ365452
*Narcine brasiliensis*	JX124815
*Narcine brasiliensis*	JX034007
*Narcine brasiliensis*	KF930170
*Pteroplatytrygon violacea*	KT307373
*Pteroplatytrygon violacea*	KT307370
*Pteroplatytrygon violacea*	KF930342
*Pteroplatytrygon violacea*	KF808209
*Pseudobatos horkelii*	JX034017
*Pseudobatos horkelii*	HM424136
*Pseudobatos horkelii*	JX034016
*Pseudobatos horkelii*	JX124879
*Rhinoptera bonasus*	JX241049
*Rhinoptera bonasus*	JX241050
*Rhinoptera bonasus*	JX241051
*Rhinoptera bonasus*	KF245598
*Rhinoptera brasiliensis*	JX124888
*Rioraja agassizii*	GU805208
*Rioraja agassizii*	GU805206
*Rioraja agassizii*	GU805204
*Rioraja agassizii*	GU805205
*Squatina guggenheim*	FN431749
*Squatina guggenheim*	FN431748
*Squatina guggenheim*	FN431747
*Squatina guggenheim*	FN431746
*Callorhinchus callorynchus*	KP719792
*Callorhinchus callorynchus*	KP719793
*Callorhinchus callorynchus*	KP719794
*Callorhinchus callorynchus*	KP719799

**Table A2:** Quantity of tons of elasmobranchs captured in the state of São Paulo, Brazil from 2012 to 2018 from the Monitoring Program for the Fishing Activity of São Paulo state (http://www.propesq.pesca.sp.gov.br/). Bottom line: the percentage of total captured by year. Final row: the percentage of total capture by fishing gear.

Fishing Gear	2012	2013	2014	2015	2016	2017	2018	Total	%
**otter-trawl**	135,026.0	98,975.8	81,566.8	82,897.1	46,071.4	20,935.4	28,360.7	493,833.0	36.34
**fish-traps**	1765.74	2457.95	1891.0	804.2	970.35	1069.9	2384.96	11,344.1	0.83
**gillnet**	112,890.0	85,540.3	70,005.7	57,010.3	91,245.7	76,519.1	88,560.2	581,771.0	42.82
**beam-trawl**	117,079.0	46,060.0	27,992.0	28,194.0	24,294.0	17,703.0	10,516.0	271,838.0	20.01
**Total**	366,761.0	233,034.0	181,456.0	168,906.0	162,581.0	116,227.0	129,822.0	1,358,786.0	100.00
**%**	26.99	17.15	13.35	12.43	11.97	8.55	9.55	100.00	
